# The Effects of Synchrony on Group Moral Hypocrisy

**DOI:** 10.3389/fpsyg.2020.544589

**Published:** 2020-12-17

**Authors:** Radim Chvaja, Radek Kundt, Martin Lang

**Affiliations:** LEVYNA Laboratory for the Experimental Research of Religion, Department for the Study of Religions, Masaryk University, Brno, Czechia

**Keywords:** group unity, moral judgment, moral hypocrisy, social bonding, synchrony, cooperation

## Abstract

Humans have evolved various social behaviors such as interpersonal motor synchrony (i.e., matching movements in time), play and sport or religious ritual that bolster group cohesion and facilitate cooperation. While important for small communities, the face-to-face nature of such technologies makes them infeasible in large-scale societies where risky cooperation between anonymous individuals must be enforced through moral judgment and, ultimately, altruistic punishment. However, the unbiased applicability of group norms is often jeopardized by moral hypocrisy, i.e., the application of moral norms in favor of closer subgroup members such as key socioeconomic partners and kin. We investigated whether social behaviors that facilitate close ties between people also promote moral hypocrisy that may hamper large-scale group functioning. We recruited 129 student subjects that either interacted with a confederate in the high synchrony or low synchrony conditions or performed movements alone. Subsequently, participants judged a moral transgression committed by the confederate toward another anonymous student. The results showed that highly synchronized participants judged the confederate’s transgression less harshly than the participants in the other two conditions and that this effect was mediated by the perception of group unity with the confederate. We argue that for synchrony to amplify group identity in large-scale societies, it needs to be properly integrated with morally compelling group symbols that accentuate the group’s overarching identity (such as in religious worship or military parade). Without such contextualization, synchrony may create bonded subgroups that amplify local preferences rather than impartial and wide application of moral norms.

## Introduction

Morality as a package of psychological and cultural adaptations has evolved to stabilize risky collective action among genetically unrelated individuals (Alexander, 1987; Greene, 2013). Group moral codes are reflected in norms that regulate access to resources, inter-personal conduct, and group defense. Breaching these norms triggers moral judgment which is reflected in a cascade of emotional responses such as anger or disgust with the delinquent and sympathy with victims (Haidt, 2013). This emotional response, in turn, motivates people to act against norm transgressors by imposing punishment for what they deem immoral behavior, thereby effectively stabilizing norm-regulated coordinative and cooperative efforts (Boyd et al., 2003; Henrich et al., 2006). In other words, moral judgment and its associated emotions serve as necessary mechanisms that facilitate group functioning by supporting normative structures that regulate social interactions.

Aside from moral judgments, cooperation in collective action is facilitated by additional social technologies,^1^ which help create in-group unity and cement group bonds between unrelated individuals, such as play and sports, dancing and music-making, or religious rituals (Kirschner and Tomasello, 2010; Tarr et al., 2016; Hobson et al., 2018). Ample research focused on disentangling the specific elements of such social technologies that facilitate the bonding effects, pointing to the positive effects of laughter (Dunbar et al., 2012), shared painful experiences (Konvalinka et al., 2011; Bastian et al., 2014), or synchronous movement and vocalization (Reddish et al., 2013b; Lang et al., 2015; Weinstein et al., 2016). Together, these bonding behaviors provide a powerful social glue that can surpass genetic relatedness (Hill et al., 2014; Whitehouse et al., 2014), effectively creating groups of committed individuals.

However, whereas morality and social bonding technologies amplify each other in small and tight-knit communities, in large-scale societies that depend on cooperation between anonymous and unrelated individuals, the relational sub-groupings created by face-to-face bonding technologies may hamper impartial application of social norms (Lang et al., 2019; Schulz et al., 2019). While breaching social norms elicits demand for punishment, the severity of this punishment and associated moral outrage may differ based on whether the transgressor is an anonymous unrelated individual, an individual from a competing subgroup, a close friend, or kin. This phenomenon, labeled as moral hypocrisy, describes the “double moral standards” that people often apply when judging others’ and one’s own behavior (Valdesolo and DeSteno, 2008; Lammers et al., 2010; Polman and Ruttan, 2012), including the discrepancy between stated prescriptions and an individual’s actual behavior (Batson et al., 1999; Batson and Thompson, 2001).

Importantly, Valdesolo and DeSteno (2007) introduced the term “group moral hypocrisy,” which describes the application of double moral standards according to group membership of the perpetrator: in their experiment, the participants judged the same moral transgression against the “next subject” less strictly when the transgressor was a member of their in-group compared to an out-group member (cf., Gneezy and Fessler, 2012; also see the discussion section for why humans sometimes punish in-groups more than out-groups). Further research showed that participants punish low offers to a third in-group player in the dictator game less strictly when the “dictator” belongs to their in-group (Bernhard et al., 2006) or that juries are more likely to enact harsher punishments when the defendants are of a different ethnicity (Hymes et al., 1993; Sommers, 2007).

The outstanding question is whether relational structuring facilitated by social technologies would also support group moral hypocrisy? In other words, if social technologies merely create locally bonded groups, these groups may fail to apply moral norms impartially and treat conspecifics preferentially when judging their moral transgressions. While there has been ample research on the positive effects of social-bonding technologies on increased cooperation and coordination on the local level (reviewed below), it is unclear whether this increased bonding also leads to moral hypocrisy, which may erode the functioning of large-scale societies comprised of anonymous individuals that rely on impartially enforced normative structures. To answer this outstanding question, we focus on one of the well-researched social bonding technologies—interpersonal motor synchrony—and investigate whether synchrony promotes moral hypocrisy of the locally bonded group.

Behavioral synchrony, i.e., matching each other’s movements in time, was shown to promote a vast array of local prosocial effects ranging from perceptual changes to cooperation. Specifically, performing synchronous movements increases perceptions of mutual similarity (Rabinowitch and Knafo-Noam, 2015), group unity (Lakens, 2010; Paladino et al., 2010; Tarr et al., 2014), social rapport (Miles et al., 2009), and sympathy between the members of the synchronized group (Hove and Risen, 2009). On the behavioral level, synchronizing with other participants elicits trust-based cooperative exchange, which translates into greater cooperation in various economic games (Wiltermuth and Heath, 2009; Fischer et al., 2013; Sullivan et al., 2015; Lang et al., 2017) and even altruistic acts in real-life situations (Valdesolo and DeSteno, 2011; Cirelli et al., 2017). For a more extensive overview of these effects, see two meta-analyses (Rennung and Göritz, 2016; Mogan et al., 2017). In summary, this research suggests that synchrony indeed strengthens social bonds at the local level, that is, between the performers.

However, there is some evidence that synchronization may also promote behaviors that may be harmful to members of other groups. For instance, compared to the asynchronous and control conditions, participants in the synchrony condition were more likely to comply with a request from another synchronized participant (confederate) to administer an unpleasant blast of noise to a member of another team (Wiltermuth, 2012). That is, participants collaborated with their synchronized partners even though the prompted behavior might be considered immoral or aggressive. Nevertheless, it is not clear how synchrony affects intra-group relations, especially in large groups where the impartial application of moral norms is crucial for the stabilization of large-scale cooperation. In other words, while there is ample evidence supporting the notion that synchrony creates local bonds, it is not clear how these local bonds affect impartial application and enforcing of moral norms and whether they support moral hypocrisy.

To this end, we designed a between-subject study where we first manipulated experienced synchrony by asking participants to engage in a movement task either with another coordination partner (high-sync and low-sync conditions) or alone in front of a blank wall. In the former conditions, the participants were asked to synchronize with another participant through a live transmission projected on a wall. This transmission was, in fact, a pre-recorded video with a confederate where the confederate was either in high-synchrony with the participants or in low-synchrony. After the synchrony manipulation, participants filled out a post-manipulation questionnaire that assessed social bonding with the confederate. Then, in an ostensibly separated session, participants were asked to help the researcher to evaluate the effectiveness of an unrelated stimulus for another study. Participants watched through a video-transmission how their synchronization partner (confederate) participates in an unrelated experiment where he commits a moral transgression against another anonymous student, and participants were asked about the fairness of such behavior.

Based on the theoretical foundations laid out above, we predicted that the participants in the high-synchrony condition would judge the moral transgression as less unfair than the participants in the low-synchrony and control conditions. Apart from this basic prediction, we also identified five potential mediators of the purported synchrony effect, namely perceived group unity, similarity, sympathy, and perceived cooperation, and explored whether moral hypocrisy is facilitated specifically by some of these mediators.

These particular mediators were chosen on the basis of their importance in the strengthening of ties between individuals as well as their previously reported association with synchrony. Therefore, these mediators were expected to facilitate the anticipated bias in the application of moral norms in the high synchrony condition. More specifically, perceived group unity should sharpen the group’s boundaries in the high synchrony condition more relative to the low synchrony and control conditions, effectively strengthening the parochial bias of norm application (Choi and Bowles, 2007). Second, similarity and sympathy are rooted in human kin psychology, providing individuals with psychological cues of genetic relatedness (Sigmund, 2009) and again strengthening the parochial bias. Finally, perceived cooperation is related to direct reciprocity (Trivers, 1971) and reputation building (Nowak and Sigmund, 2005). The positive effects of cooperation should, therefore, affect nepotistic cooperation also in other contexts, namely during moral judgment. Investigating these mediators formed an exploratory part of the current study that should suggest venues for future research.

## Materials and Methods

### Participants

Using a sample size from similar studies (e.g., Wiltermuth and Heath, 2009; Reddish et al., 2016; Lang et al., 2017), we recruited 129 participants (82 females, *M*_age_ = 23.1, SD = 5.4) from the student pool at Masaryk University (subjects participated in exchange for course points which they needed to complete the course) and randomly assigned them to one of the three conditions: high synchrony (16 males, 25 females), low synchrony (18 males, 31 females), and control (13 males, 26 females). Four subjects expressed doubts about the authenticity of the video transmission at the end of the experiment. We decided to retain their data in the analyses presented in the main text because removing the data does not qualitatively affect the results as we show in the Supplementary Material (SM),^2^ Section S1. All subjects were debriefed after the end of the data collection.

### Materials

To manipulate synchrony, we utilized the general procedure and specific videos from Lang et al. (2017). Participants engaged in two^3^ 5-min rounds of motor activity to induce the differential levels of synchrony. In the high and low synchrony conditions, the participants were asked to perform a motor task together with a second participant who was located in another room through a live video transmission. The participants in both synchrony conditions were instructed to perform easy hand-movement sequences with a gong sound announcing the start of each movement sequence, and to synchronize those movements with the movements of another participant. In reality, the transmission was a pre-recorded video (see the videos in SM, see footnote text 2), which was designed to accurately manipulate the participants’ experience of either high or low synchrony.

To increase the feeling that the video was a real-time transmission, we added a loading sequence to the beginning of the video consisting of a loading symbol and text stating “waiting for the connection” and “waiting for the other party.” The confederate in the pre-recorded video was a male in his thirties with a gray square covering his face to reduce the influence of attractiveness and sympathy (for more details, see Lang et al., 2017). In the high synchrony condition, the confederate made no mistakes during the task. In the low synchrony condition, the confederate made systematic errors: (1) the speed of the confederate’s hand movements varied; (2) the confederate’s reaction time was delayed by 0.1, 0.3, and 0.5 s; and (3) the confederate performed different movements 15 times during each round. In the control (baseline) condition, the participants were instructed to perform the same hand-movement sequences alone in front of a blank canvas (this served as a projection canvas in the other conditions). The control condition was included as a baseline measure to assess whether the potential difference between the two synchrony conditions might be caused by the high synchrony making the moral judgment more lenient or by the low synchrony making the moral judgment harsher.

### Measurements

Immediately after this moving task we measured several single-item and multi-item variables such as potential mediators of the hypothesized effect of synchrony on moral hypocrisy and manipulation checks (see the Post-Manipulation Questionnaire in Section S2 in SM, see footnote text 2). These latent variables included: (1) perceived synchronization with the confederate (five items, Cronbach’s α = 0.90) as a manipulation check^4^; (2) perceived cooperation (six items, Cronbach’s α = 0.90), sympathy (three items, Cronbach’s α = 0.88), similarity (two items, split-half reliability = 0.81), and group unity (three items, Cronbach’s α = 0.84) as potential mediators; and (3) mood (six items, Cronbach’s α = 0.86) and the physical and psychological difficulty of the synchronization task (single-item variables) as control variables. All questions were answered on nine-point Likert scales (1 = not at all, 9 = yes, definitely). When answering these questions, participants in the control condition were instructed to imagine a random person from the participant pool and relate their answers to that person. In doing so, participants in the control condition were also exposed to some level of cognitive load similarly as the participants in the synchrony conditions. While participants in the synchrony conditions had to pay attention to the movements of the confederate, participants in the control condition had to mentally project a third person on the wall.

To assess moral hypocrisy, we used a modified version of the task utilized by Valdesolo and DeSteno (2007). Participants were informed that a colleague of the researcher running the current experiment needs feedback on their newly developed application for assigning participants into different conditions. The participants’ task was to assess the functionality of the application by watching (via “shared screens”) the confederate with whom they have previously synchronized. The piloted study’s title was “The impact of music on analytical thinking” and the video was again pre-recorded. It started with the same loading sequence as the synchrony task video, and then proceeded with the synchrony partner answering a few questions related to the musical record (e.g., “Did you find the record boring?”) to induce the feeling that the application was being tested in real time. The authenticity of the video was also reinforced by adding the moving confederate’s cursor to the screen. After the cursor showed confederate’s answers to these questions, the application instructed the confederate to use a randomizer that would allocate the following task either to himself or to the next subject in the experiment. The other subject was described simply as a “next participant” without any further specification to standardize any biases participants might have had. Nevertheless, participants knew that only students from the course may participate in the experiments which implicitly formed the wider in-group of students from the same university. There were two types of tasks allocated: an easy green task containing simple mathematical exercises and lasting approximately 10 min, or a difficult red task full of mathematical equations that would take approximately 40 min.^5^ In the video, the confederate used the randomizer and was assigned the difficult task but nonetheless selected the easy task on purpose and left the difficult task to the next participant.

After seeing this video (which they were led to believe was a live video transmission), the participants completed a pen-and-paper^6^ questionnaire with several distracting questions regarding the quality of the application. Mixed within these questions was our main dependent variable, a question that assessed the fairness of the confederate’s behavior: “Did the participant act fairly?” (1 = not at all, 9 = very much). Note that we chose to ask about fairness because directly mentioning morality would not fit the cover story (see Section S3 in SM for the Feedback Questionnaire, see footnote text 2).

### Procedure

After reading the information about the study and signing informed consent, participants were asked to perform two rounds of simple motor activity. All the procedural steps were explained via a pre-programmed application (written in HTML and run online via an internet browser) on a computer that was connected to the online post-manipulation questionnaire. The instructions presented in the application first described the whole procedure, stating that there will be two 5-min rounds of easy hand movements. The participants in the synchrony conditions were told that they will perform these movements together with another participant in a different room while the participants in the control condition were told only that there is another participant doing the same task in a different room. Afterward, the instructions provided participants with a precise description of the first-round of movements accompanied by pictures with our colleague showing how to perform all movements step-by-step. Participants were then given free time to practice. The same procedure followed after the first round, although the movements were slightly different so participants would not get bored. After the second round, the instructions redirected participants to a post-manipulation questionnaire that assessed the mediating variables explained above. After filling out the post-manipulation questionnaire, participants were instructed to knock on the door of an adjacent room. Then, a research assistant invited participants to take part in a pilot testing of another experiment and asked them to give feedback on this procedure, which was currently under development. Participants then watched the video with moral transgression and subsequently received a pen and paper with feedback questions. In the final step, participants were asked about their suspicion of the goals of the current experiment (“What do you think the whole experiment was about?”) and were thanked for their participation. The whole procedure took about 50 min.

### Analysis

All analyses were performed in R, version 3.5.3 (R Core Team, 2019). To analyze the differences in moral judgment (and also other variables) that were recorded as on a 9-point Likert scale, we fitted linear regression models. Only linear models comprising the manipulation-check variables of physical and mental difficulty as dependent variables revealed non-normally distributed residuals. Thus, we re-analyzed these variables using cumulative link models from the package *ordinal* (Christensen, 2019) that are suitable for modeling ordinal data. SM Section S4 (see footnote text 2) includes residual diagnostics of the two linear models with poor fit and also the fit diagnostics of the main model with moral judgment. The Supplementary R code includes residual diagnostics for all models in this manuscript. All fitted models were adjusted for sex because women and men were not distributed evenly across the different conditions. Since all of the self-reported variables were measured on nine-point Likert scales, we report simple effect sizes (unstandardized regression coefficients) rather than standardized effect sizes (Baguley, 2009). For cumulative link models, we also report odds ratios in the table. The mediation analysis was performed using the function *sem* from package *lavaan* (Rosseel, 2012). Table 1 displays the estimated differences between conditions.

**TABLE 1:** Unstandardized regression slopes with standard errors for the effects of condition on manipulation check variables, moral judgment, and potential mediators.

	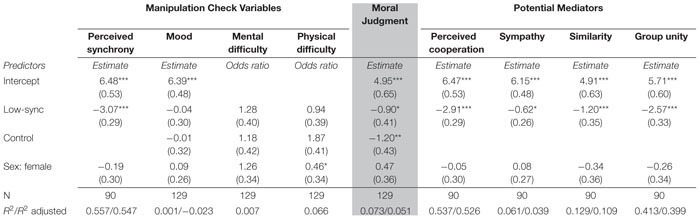			

Each column represents individual dependent variable while rows show the intercepts (set as the high synchrony condition), and estimated differences between the intercept and the low-synchrony and control conditions. That is, the slopes indicate by how much the dependent variable shifts (on a nine-point Likert scale) when going from the intercept to the low-synchrony and control conditions. For sex, males are the reference category and the estimate is the difference between males and females. Mental difficulty and Physical difficulty were analyzed using cumulative link models. R^2^ for these two models represents Nagelkerke pseudo R^2^. Moreover, function clm that we used for cumulative link models does not compute intercepts; thus, we do not display intercepts for those two models. The perceived synchrony and mediator models include reduced sample size because they exclude the control condition that had no interacting partner.* p < 0.05; ** p < 0.01; *** p < 0.001.

## Results

### Manipulation Check

To assess whether our manipulation was effective in eliciting differential synchrony levels, we first regressed the perceived synchrony construct on our conditions. Adjusting the model for sex, the participants in the low-sync condition indicated lower levels of perceived synchrony than those in the high-sync condition (*b* = −3.07, 95% CI [−3.65, −2.48]). Note that because participants in the control condition performed movements alone, we did not include this condition in the analysis of the manipulation check here. However, this analysis was recommended by one of the reviewers in Frontiers in Psychology (see SM Section S5, see footnote text 2).

Furthermore, the mood and perceived physical and mental difficulty of the task was not predicted by the condition. Neither the low-sync condition (*b* = −0.04, 95% CI [−0.64, 0.56]) nor the control condition (*b* = −0.01, 95% CI [−0.64, 0.63]) were associated with statistically reliable differences in mood compared to the high-sync condition. The same absence of difference was observed for perceived physical difficulty (low-sync condition: *b* = −0.06, 95% CI [−0.83, 0.72]; control condition: *b* = 0.63, 95% CI [−0.17, 1.43]) and mental difficulty (low-sync condition: *b* = 0.25, 95% CI [−0.54, 1.04]; control condition: *b* = 0.17, 95% CI [−0.66, 1.00]). Interestingly, women reported that the task was less physically demanding than the men.

### Main Analysis

**FIGURE 1:**
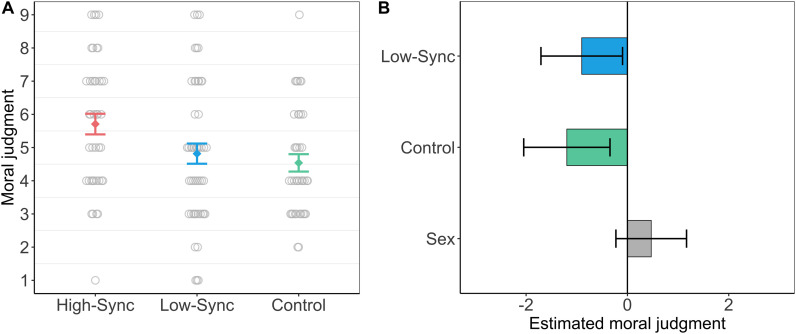
**(A)** Displays the raw differences in moral judgment between conditions with 95% CI. **(B)** Displays the estimated differences from the intercept (the high-synchrony condition) for the low-synchrony condition and the control condition with 95% CI. Sex shows the difference between males (the intercept) and females.

Next, we analyzed the main effect of the condition on moral judgment. The average moral judgment located on a 1–9 scale and anchored by “totally unfair” and “totally fair” was 5.70 (*SD* = 2.00) in the high-sync condition, 4.82 (*SD* = 2.13) in the low-sync condition, and 4.54 (*SD* = 1.64) in the control condition. These differences also showed a stable pattern in the linear regression framework: compared to the high-sync condition, the low-sync condition was associated with the lower fairness rating (*b* = −0.90, 95% CI [−1.71, −0.09]), as was the control condition (*b* = −1.20, 95% CI [−2.06, −0.34]). See Figure 1A for raw differences and Figure 1B for estimated differences between conditions.

### Exploratory Mediation Analysis

After detecting the effect of the condition on moral judgment, we proceeded with the mediation analysis, where we first assessed whether the potential mediators were affected by the synchrony treatment. Results displayed in Table 1 show that all potential mediators were affected by our manipulation. Then, we used AIC model selection to decide which potential mediator variable should be modeled as a mediator using structural equation modeling (SEM). Note that the potential mediators were meaningful only in the high and low synchrony conditions. Thus, we used only data from these two conditions for all the following analyses. We built five models which are displayed in Table 2. First model is a reference model with sex as a single predictor. The following four models include respective mediators together with sex. The predictor from the model that has AIC value at least two points lower than the reference model was chosen to be modeled as a mediator using SEM. AIC numbers displayed in Table 2 suggest that the only suitable variable is perceived group unity between participants and the confederate (the difference between AICs of these models is 4.49).

**TABLE 2:** The association between potential mediators and moral judgment with 95% confidence intervals.

	**Reference Model**	**(1)**	**(2)**	**(3)**	**(4)**
*Predictors*	*Estimate*	*Estimate*	*Estimate*	*Estimate*	*Estimate*
Intercept	4.34*** (2.80-5.87)	3.13*** (1.36-4.89)	3.37*** (1.50-5.24)	4.87*** (2.99-6.75)	3.40** (0.85-5.95)
Sex: female	0.55 (−0.36-1.45)	0.63 (−0.25-1.52)	0.57 (−0.33-1.47)	0.50 (−0.41-1.41)	0.54 (−0.37-1.44)
Group unity		0.27* (0.06-0.49)			
Cooperation			0.19 (−0.03-0.41)		
Sympathy				−0.12 (−0.38-0.13)	
Similarity					0.16 (−0.19-0.51)

Observations	90	90	90	90	90
*R*^2^/*R*^2^ adjusted	0.016/0.005	0.084/0.063	0.050/0.028	0.027/0.004	0.025/0.003
AIC	392.741	388.252	391.621	393.768	393.876

Note that the AICs of models 2–4 are not substantially lower than the reference model which means that these models are not substantially more informative than the reference model.*p < 0.05; **p < 0.01; ***p < 0.001.

**FIGURE 2:**
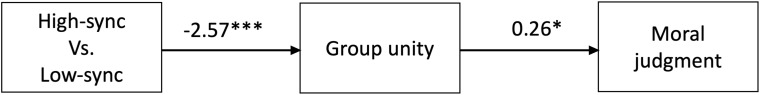
The mediation model. Note that the estimated slopes are different from those displayed in Table 2 because we did not included sex as covariate in the mediation analysis. Note: ^∗^*p* < 0.05, ^∗∗∗^*p* < 0.001.

Therefore, we built a structural equation model with group unity as a mediator. The model showed good fit to the data even when stringent cut-off values were used given the sample size of the present study (RMSEA = 0.00, SRMR = 0.02, CFI = 1.00, TLI = 1.03; see Sivo et al., 2006). The specific estimates from this mediation model are reported in Figure 2.

## Discussion

In this study, we tested whether interpersonal motor synchrony affects participants’ judgment of moral transgression committed by their synchrony partner against another anonymous non-synchronized person. Moreover, we also explored whether this effect would be mediated by psychological mechanisms synchrony is known to promote, namely group unity, perceived cooperation, similarity, and sympathy. We found that participants in the high-synchrony condition judged the same moral transgression committed by their synchrony partner as less unfair than participants in the low-synchrony and control condition. The results of the mediation analysis suggested that perceived group unity mediated the effect of synchrony on moral judgment, i.e., feeling more united with the synchronized partner led to more lenient judgments.

The results of the present study suggest that a society-wide application of cultural norms may be hampered by social bonding technologies that modify moral judgment based on the perpetrator’s sub-group identity. By creating smaller compact groupings within the larger society, bonding technologies such as synchrony may motivate preferential treatment of the bonded partners. We conjecture that this effect is akin to the real-world phenomena such as cronyism and nepotism when a society’s resources are preferentially distributed along kith and kin lines (Schulz et al., 2019). While the present study focused specifically on the effects of synchrony on group moral hypocrisy, we expect that similar effects should be observed with other bonding technologies such as extreme rituals (Xygalatas et al., 2013) and political rallies (McNeill, 1995).

Social bonding technologies have been instrumental in the functioning of smaller communities as they facilitate tribal morality (Fischer and Xygalatas, 2014), which is crucial in competition between different groups (Bowles, 2006; Choi and Bowles, 2007). However, the same bonding technologies may be detrimental to the functioning of large-scale societies, unless these technologies are properly integrated within mechanisms that support society-wide norms such as moralizing gods (Purzycki et al., 2016; Lang et al., 2019), state ideologies (McNeill, 1995), and social institutions (Gaechter and Schulz, 2016). While social bonding technologies such as dance or collective rituals likely evolved to facilitate group cohesion within smaller communities of nomadic hunter-gatherers (to promote tribal morality Lang, 2019), these technologies could be scaled up to support larger societies with hierarchical leadership structure by emotionally charging universally shared symbols, norms and institutions (Alcorta and Sosis, 2005; Fischer and Kruekaew, 2019). That is, with appropriate group hierarchy and shared symbols, the locally confined bonding effects of synchrony might be scaled to the society level (e.g., local parishes facilitating adherence to the Roman Catholic Church), effectively supporting an overarching group identity and impartial norm application (Lang et al., 2019); but without such established hierarchy, synchrony may promote biased application of moral norms.

If the conjecture that synchrony supports moral tribalism is correct, then it should be expected that the results of the current study would change according to the perpetrator’s and victim’s identities. In the current study, participants were informed that their synchrony partner (transgressor) and the third participant (victim) are subjects from the same pool, implying that they are both part of participants’ extended in-group; i.e., all of them are students in the same class (but anonymous to each other). Despite this weakly shared general identity, synchrony led to the hypocritical judgment that downplayed the maltreatment of an anonymous student from an extended in-group. Since Wiltermuth (2012) showed that synchrony is also conducive to harming out-group members, we expect that synchrony should facilitate more lenient (and perhaps even approving) judgment of norm transgressions against out-groups. It is safe to assume that if the victim would be of more distant identity such as a student from a different university, we should expect even stronger effects of synchrony on hypocrisy. Indeed, previous cross-cultural research showed that morality is highly parochial and often does not extend beyond group borders (Fessler et al., 2015), unless promoted as a conversion strategy to attract more people into the group (Lang et al., 2019).

Furthermore, if all three actors in the current study would assume the same tightly shared identity such as membership of a soccer hooligans fan club or of a gang, the synchrony effects on moral hypocrisy should disappear or even reverse. This prediction is supported by the black sheep hypothesis, which argues that individuals apply harsher judgments to inappropriate behavior of in-groups because the harmful effects of norm-transgression trickle down, by extension, to all group members (Marques et al., 1988). The reverse effects of synchrony should pan out especially during inter-group conflict when adherence to widely shared norms might be the key factor influencing success in intergroup competition (Richerson et al., 2016). In support of this prediction, Gneezy and Fessler (2012) documented increased altruistic punishment of in-group norm transgressors during the 2006 Israel–Hezbollah war, and inter-group conflict was shown to bolster equality and equality-promoting norms long after the ceasefire (Bauer et al., 2014; Henrich et al., 2019).

An alternative to the proposed tribalistic effects of synchrony is the generalized prosociality hypothesis, formulated around the empirical findings of Reddish and colleagues (Reddish et al., 2013a, 2016). In their experimental study of the relationship between synchrony and prosociality, Reddish et al. (2013a) found that the synchronized participants were more willing to help another student who did not synchronize with them and sent a larger portion of their monetary endowment to members of an out-group (compared to participants who did a puzzle task together). In another study, Reddish et al. (2016) found that the synchronized (vs. asynchronized) participants were more willing to help an anonymous out-group student whereas there was no difference in willingness to help a member of an extended in-group. These results are in contrast with the present study where we found that, at least in the moral domain, synchrony effects are selective rather than generalized (see also Wiltermuth, 2012). If synchrony would produce generalized prosociality, we should expect greater prosociality toward the victim and harsher judgment of the transgressor in the synchrony condition; or no effect of synchrony at all, depending on the strength of the assumed prosociality. What may account for the differences between the current study and the studies by Reddish et al. (2013a, 2016)?

One possible explanation may be the different manipulations employed across these studies. For instance, while Reddish et al. (2016) let groups of three or four people synchronize (or move in asynchrony) together in the laboratory, our participants were alone in the room and all interactions with the confederate was mediated through a video-transmission. Although a previous study using video-transmission found sizable effects of synchrony on cooperation (Lang et al., 2017), we do not know whether the lack of other people in the room may inhibit the effects of synchronization on generalized prosociality. Furthermore, the generalized prosociality effects of synchrony may be specific to the dependent variables assessed in those studies. While synchrony positively affected monetary contributions to out-groups in Reddish et al. (2013a), the same study failed to find any effects of synchrony on mitigating self-reported preferential biases toward in-groups rather than out-groups. Yet another explanation of these disparate findings could be that synchrony positively affects moral hypocrisy independently of the transgressor’s identity (akin to the generalized effects on prosociality). That is, synchronization may reduce moral vigilance such that synchronized participants would judge any norm transgressions as less severe, no matter who committed them. Although this hypothesis requires proper experimental testing, the results of our mediation analysis suggest that this is likely not to be the case.

The mediation analysis revealed that the only mediator was perceived group unity. In the current study, rather than having generalized effects, synchrony affected moral judgment by strengthening the unity between the synchronized partners. As a consequence, the perceived group boundaries between the participant and the victim were sharpened, even though both were members of an extended in-group (students from the same university). Indirect support for this speculation could be drawn from research on the effects of entitativity on prejudices toward out-groups (Gaertner and Schopler, 1998): perceiving one own’s group as an entity both correlates with and fosters out-group prejudice (Effron and Knowles, 2015). Nevertheless, this conclusion does not yet explicate why sympathy and similarity were not mediators of the synchrony effect on moral judgment, despite their direct connection to the process of group formation. That is, why did similarity and sympathy not mediate the effects of synchrony on group moral hypocrisy?

The perceptions of similarity and sympathy to others are tightly interwoven and provide subtle cues on genetic kinship (Sigmund, 2009), with objective or even perceived similarity having positive effects on cooperation in economic games and in leader-follower interactions (Krupp et al., 2008; Cornelis et al., 2011). Perhaps, the increased feelings of similarity and sympathy induced by synchrony may have spilled over to the third participant (the victim), and participants also felt closer to the victim, effectively creating a group that included both the synchronized partner and the victim. (Note that participants were first asked about sympathy and similarity to the sync partner and only afterward observed his immoral behavior; hence, they were forced to explicitly think about similarity and sympathy before the transgression task took place). In fact, Table 2 suggests that similarity was negatively associated with moral judgment, although the effect was not precisely estimated. Returning to the example of inter-group conflict, perceiving that all group members are committed to the same cause (unity) directed against another group, should be sufficient motivation for taking part in the clash, even without necessarily being close (similarity, sympathy) to the other members of the hooligan fan club. Moreover, by emphasizing group boundaries, harming members of the other group might appear as permitted and even desired (cf., Newson et al., 2018).

The final variable with mediating potential that we evaluated, perceived cooperation during the synchronization task, positively predicted moral hypocrisy, although this effect was less precisely estimated than the effect of group unity. Moreover, adding perceived cooperation to the model did not increase the explanatory power of the model compared to the reference model with sex as a single predictor. Whereas perceived cooperation was previously implied as an important mediator of the synchrony effects on prosociality (Reddish et al., 2013b but see Lang et al., 2017), it may have weaker effects on moral judgment because perceived cooperation does not directly influence perceived group boundaries. Nevertheless, the small predictive power of perceived cooperation that we observed might be attributed to the principle of reciprocity (Trivers, 1971). Similar to the effects on prosocial behavior, where perceived cooperation increased the probability that synchronized partners will also cooperate in the following interactions, the participants who scored higher on perceived cooperation may have felt obliged to help their partner (the transgressor) as a form of reciprocal exchange for preceding successful coordination. As the mediation analysis was only exploratory, future research should test the causal effects of the group unity on group moral hypocrisy. Such an experiment may directly manipulate the perceived group unity and then use the task we used to measure moral hypocrisy.

Apart from the direct test of mediators, we propose that further important insights into the suggested social bonding effects of synchrony might be gained by examining the neuro-hormonal underpinnings of synchrony. For example, while a lot of attention has been paid to the understanding of the β-endorphin‘s role in mediating prosocial effects of synchrony (Cohen et al., 2010; Sullivan et al., 2014; Tarr et al., 2015, 2017; Launay et al., 2016; Weinstein et al., 2016; Lang et al., 2017), substantially less attention has been devoted to another important social hormone, namely oxytocin. In fact, we are aware of only one study that showed increased levels of oxytocin in reaction to a group singing lesson across professionals and amateurs (Grape et al., 2003) and only one study (Gebauer et al., 2016) that investigated whether oxytocin promotes synchronization. Since oxytocin is often considered to be a parochial hormone that promotes positive behavior toward family members (Galbally et al., 2011) but negative attitudes (De Dreu et al., 2011) and behavior toward out-group members (Zhang et al., 2019), studying whether synchrony increases oxytocin levels is the next logical step in the future synchrony research.

Likewise, to achieve better generalizability and higher validity, the examination of synchrony effects on moral group hypocrisy should move to real environments such as collective religious rituals (e.g., Xygalatas et al., 2013) or football stadiums (e.g., Newson et al., 2018). The real-life conditions might either foster or diminish the laboratory-induced effects of synchrony, depending on other socio-cultural factors such as religious identities (Purzycki et al., 2016; Lang et al., 2019) or the presence or absence of intergroup conflict (Bauer et al., 2016). Moreover, if synchrony evolved as a social technology that helped cement bonds within small communities of nomadic hunter-gatherers during inter-group competition (Choi and Bowles, 2007), we should expect the frequency of group synchronous practices to increase during inter-group conflict, similar to other mechanisms promoting norm adherence (Sosis et al., 2007; Schaub, 2017; Francois et al., 2018; Henrich et al., 2019). This prediction could be tested against data from ethnographic and historical databases, large-scale surveys, or in the laboratory (Miles et al., 2011).

Finally, another extension improving the limitations of the current study would be to measure the ultimate behavioral outcome of moral judgment, namely altruistic punishment (Boyd et al., 2003; Fehr and Fischbacher, 2003) or, alternatively, an action to stop the moral transgression. While we aimed to increase the validity of the current study by adopting a real-world scenario of norm transgression instead of just using vignettes with hypothetical moral transgressions, such a scenario only allowed us to measure moral judgment, which is usually cheap to produce (cf., Saltzstein, 1994). Moral judgment might motivate others to adhere to norms due to reputational sensitivity, however, it remains to be tested whether synchrony also affects more lenient punishments. Therefore, we suggest that future studies should adopt different and more nuanced behavioral measures such as altruistic punishment, helping the victim of the transgressions, or the tendency to copy the immoral behavior.

## Data Availability Statement

The dataset generated in this study can be found in online repository at: osf.io/pfu6e.

## Ethics Statement

The study was approved by the Research Ethics Committee at Masaryk University. Participants provided their written consent.

## Author Contributions

RC and RK developed the research idea. RC designed the study with contributions provided by RK and ML. RC managed the data collection. RC and ML analyzed the data and drafted the manuscript with revision provided by RK. All authors approved the final manuscript.

## Conflict of Interest

The authors declare that the research was conducted in the absence of any commercial or financial relationships that could be construed as a potential conflict of interest.

## Footnotes

1 We are using the term “social technology“ in the same way as Fischer and Xygalatas (2014) to refer to behavioral patterns deeply rooted in human evolutionary history that provide solutions to the problem of cooperation. Note that these behaviors operate on a bio-social level. For example, religious rituals are transmitted socially but they activate certain biological and psychological reactions that affect human cooperation (e.g., Charles et al., 2020).

2 SM is accessible at osf.io/pfu6e. This link contains raw data in the *.xlsx* format, R scripts in the.*html* format, two examples of video stimuli, and SM document in the *.pdf* format. In the *.pdf* SM document, sections S2 and S3 list survey questions used to obtain control, check, mediation and dependent variables while sections S1, S4, and S5 contain supplementary analyses that are not displayed in the main text.

3 While Lang et al. (2017) used three rounds of the motor task, we opted to use only two rounds. Lang et al. (2017) were interested in measuring pain threshold as a proxy of β-endorphin release after each round of the coordination task and, therefore, needed more rounds.

4 Note that Lang et al. (2017) demonstrated the validity of this manipulation by both directly asking the participants about perceived synchrony and by analyzing the temporal synchronization of participants’ hand movements with the video using accelerometers positioned on participants’ wrists (i.e., measuring arm movement acceleration). This additional measurement allowed Lang et al. (2017) to assess the extent of actual movement synchronization between participants and the confederate in the high and low sync condition, showing that participants in the high sync condition had no problems to match the confederate’s movements (made no mistakes) while the movements in the low sync condition were misplaced mostly due to the confederate’s mistakes. To assure that our manipulation had similar validity, we included at least a question about perceived synchronization.

5 During our pilot study, we utilized the original version of the task used by Valdesolo and DeSteno (2007). In this first version, the confederate was only recommended to use the randomizer with the sentence “participants usually use the randomizer.” However, this was not a direct instruction. In the first pilot testing (*n* = 22) of this version of the task, participants were asked to judge the behavior on a dichotomous scale (Yes/No), then on a nine-point scale, and finally to provide a reason for their decision. Pilot participants often reported that the behavior of the confederate was not unfair because using the randomizer was not obligatory. Thus, we changed the instruction in the video as follows: “For fair assignment, use our randomizer that randomly assigns you the color of the condition that you should be part of.” After this modification, we again piloted the new task (*n* = 9). Participants in this second pilot did not report similar concerns as participants in the first pilot; therefore, we used this version for the present experiment.

6 The reason to use a paper questionnaire instead of an online survey was to harness all potential means to induce the feeling that providing feedback on the application really was separate from the original experiment where participants answered online surveys.
